# Comprehensive molecular analyses of cuproptosis-related genes with regard to prognosis, immune landscape, and response to immune checkpoint blockers in lung adenocarcinoma

**DOI:** 10.1007/s00432-024-05774-7

**Published:** 2024-05-09

**Authors:** Ruixia Li, Run Tong, Jasmine Lin Zhang, Zhe Zhang, Mingming Deng, Gang Hou

**Affiliations:** 1 Department of Pulmonary and Critical Care Medicine, Second Affiliated Hospital of Harbin Medical University, Harbin, China; 2 National Center for Respiratory Medicine, Chinese Academy of Medical Sciences, Beijing, People’s Republic of China; 3 American International School, Hong Kong, People’s Republic of China; 4 Department of Pathology, Shengjing Hospital of China Medical University, Shenyang, People’s Republic of China; 5 National Clinical Research Center for Respiratory Diseases, Chinese Academy of Medical Sciences, Beijing, People’s Republic of China; 6 Institute of Respiratory Medicine, Chinese Academy of Medical Sciences, Beijing, People’s Republic of China; 7 Department of Pulmonary and Critical Care Medicine, Center of Respiratory Medicine, China-Japan Friendship Hospital, Beijing, People’s Republic of China; 8 State Key Laboratory of Respiratory Health and Multimorbidity, Beijing, People’s Republic of China

**Keywords:** Lung adenocarcinoma, Cuproptosis, Cell death, Immune profile, Immunotherapy

## Abstract

**Background:**

Recent studies have emphasized the importance of the biological processes of different forms of cell death in tumor heterogeneity and anti-tumor immunity. Nonetheless, the relationship between cuproptosis and lung adenocarcinoma (LUAD) remains largely unexplored.

**Methods:**

Data for 793 LUAD samples and 59 normal lung tissues obtained from TCGA-LUAD cohort GEO datasets were used in this study. A total of 165 LUAD tissue samples and paired normal lung tissue samples obtained from our hospital were used to verify the prognostic value of dihydrolipoamide S-acetyltransferase (DLAT) and dihydrolipoamide branched chain transacylase E2 (DBT) for LUAD. The cuproptosis-related molecular patterns of LUAD were identified using consensus molecular clustering. Recursive feature elimination with random forest and a tenfold cross-validation method was applied to construct the cuproptosis score (CPS) for LUAD.

**Results:**

Bioinformatic and immunohistochemistry (IHC) analyses revealed that 13 core genes of cuproptosis were all significantly elevated in LUAD tissues, among which DBT and DLAT were associated with poor prognosis (DLAT, HR = 6.103; DBT, HR = 4.985). Based on the expression pattern of the 13 genes, two distinct cuproptosis-related patterns have been observed in LUAD: cluster 2 which has a relatively higher level of cuproptosis was characterized by immunological ignorance; conversely, cluster 1 which has a relatively lower level of cuproptosis is characterized by TILs infiltration and anti-tumor response. Finally, a scoring scheme termed the CPS was established to quantify the cuproptosis-related pattern and predict the prognosis and the response to immune checkpoint blockers of each individual patient with LUAD.

**Conclusion:**

Cuproptosis was found to influence tumor microenvironment (TME) characteristics and heterogeneity in LUAD. Patients with a lower CPS had a relatively better prognosis, more abundant immune infiltration in the TME, and an enhanced response to immune checkpoint inhibitors.

**Supplementary Information:**

The online version contains supplementary material available at 10.1007/s00432-024-05774-7.

## Introduction

Lung cancer remains the most common cause of cancer-related deaths worldwide, and its morbidity continues to increase (Brody 2020). The five-year survival rate is only approximately 20% because of its asymptomatic features and lack of efficient screening methods (Nasim et al. 2019; Wu et al. 2021). Lung adenocarcinoma (LUAD), the major histological subtype of lung cancer, poses an enormous challenge for Patients with LUAD and physicians. Traditional treatments, including surgical resection, chemotherapy, and radiotherapy, are widely used. The emergence of molecular targeted therapies and immunotherapies has created more opportunities for patients with LUAD. Although improvements have been made in LUAD treatment, the clinical benefits remain modest because of immune resistance and the molecular heterogeneity of LUAD (de Sousa and Carvalho 2018; Spella and Stathopoulos 2021). Thus, it is important to identify novel therapeutic strategies, clear molecular subtypes, and robust prognostic signatures for LUAD.

Recently, the relationship between different forms of cell death and tumors has been explored in various solid tumors (Denisenko et al. 2018). To identify more effective potential treatment therapies for LUAD, numerous studies have explored the complex relationship between various types of cell death and LUAD. Based on cell death-related genes, prognostic signatures for patients with LUAD have been used to predict overall survival (Zhao et al. 2020; Gao et al. 2021; Lin et al. 2021). With regard to ferroptosis, knocking down the cAMP response element-binding protein, which can further promote ferroptosis-like cell death, has been verified as a promising strategy for treating LUAD; blocking exosomes to sensitize LUAD cells to ferroptosis has also been proven in in vivo models and is regarded as a ferroptosis-based treatment (Wang et al. 2021; Zhang et al. 2022). Regarding apoptosis in LUAD, single-cell sequencing data revealed that there was a relatively higher expression level of anti-apoptosis-related genes in LUAD tissues, and there were drugs that exhibited inhibitory effects on LUAD by inducing apoptosis and autography (He et al. 2021; Zhang et al. 2021a, b). Necroptosis was found to be an independent risk factor for patients with early stage LUAD (Oiwa et al. 2021). Cuproptosis, a newly reported form of regulated cell death, has attracted considerable attention in the recent years. Unlike the known forms of cell death such as ferroptosis and pyroptosis, cuproptosis is triggered by mitochondrial stress instead of oxidative stress. This process is triggered by relatively high concentrations of intracellular cupper (Cu) (Tsvetkov et al. 2022). Whether there is a connection between cuproptosis and tumor immunity in LUAD and how cuproptosis contributes to the heterogeneity of LUAD remains to be clarified. Therefore, by reviewing recent studies on cuproptosis, we identified 13 cuproptosis-related genes, which we considered a breakthrough point to explore the relationship between cuproptosis and LUAD.

In our study, based on cuproptosis-related genes, 497 patients with LUAD in the TCGA-LUAD cohort were divided into two subtypes. Discrepancies in immune cell infiltration and drug resistance were also investigated. A cuproptosis-related scoring scheme was established to quantify the cuproptosis patterns and predict the prognosis in patients with LUAD. The findings reported herein suggest that cuproptosis plays an important role in shaping the immune infiltration profiles of LUAD, and can help guide individualized therapies.

## Materials and methods

### Collection and preprocessing of publicly attainable LUAD datasets

The mRNA expression data and relevant clinical features of 852 samples, including 793 LUAD samples and 59 normal lung tissues, were gathered in this study under the criteria of explicit expression profiles and detailed follow-up information. Gene expression profiles in the form of fragments per kilobase of transcript per million fragments mapped (FPKM), clinical features, and mutation information of 497 LUAD samples and 59 normal lung tissues were downloaded from The Cancer Genome Atlas (TCGA) data portal (https://portal.gdc.cancer.gov/) and employed as the training set for its optimal size. The training set contained three independent LUAD datasets acquired from the Gene Expression Omnibus database (https://www.ncbi.nlm.nih.gov/geo/) under the accession numbers GSE19188 (N = 40), GSE31210 (N = 226), and GSE29013 (N = 30) in the form of processed series matrix files.

### Patients and tissue samples

For a total of 165 cases, LUAD tissue and paired normal lung tissue samples were obtained from the Shengjing Hospital of China Medical University(2023PS806K). The clinical characteristics of each patient with LUAD, including age, age at initial diagnosis, and stage at diagnosis (tumor, node status, metastasis, and TNM classification), were obtained from medical records and pathological reports (Supplementary Table 5). The inclusion criteria were as follows: (1) presence of a single lung nodule; (2) histological diagnosis of lung adenocarcinoma (LUAD); (3) no prior history of other malignant tumors; (4) patients with primary, treatment-naive LUAD. This study was approved by the Human Ethics Review Committee of the Shengjing Hospital of China Medical University.

### Immunohistochemistry (IHC) analysis

IHC staining was performed as previously reported (Deng et al. 2020a, b, c). Dihydrolipoamide branched chain transacylase E2 (DBT) polyclonal antibody (12,451-1-AP; Proteintech) and dihydrolipoamide S-acetyltransferase (DLAT) polyclonal antibody (13,426-1-AP; Proteintech) were used as the primary antibodies. Phosphate-buffered saline was used as a negative control. Each section was evaluated and scored independently by two pathologists. A semi-quantitative scoring system was used for this assay. The intensity was scored as follows: 0, negative; 1, weak; 2, moderate; 3, strong. We also calculated the proportion of tumor cells within each category. The proportional score was multiplied by the staining intensity score to obtain the final immunohistochemistry (IHC) score. The IHC scores ranged from 0 (minimum) to 300 (maximum). Positive expression was defined as a detectable immunoreaction with an IHC score > 10.

### Consensus molecular clustering of 13 cuproptosis-related genes by ConsensusClusterPlus

We retrieved literature relevant to cuproptosis, and a total of 13 recognized genes were selected and applied to identify distinct cuproptosis-related patterns in LUAD (Tang et al. 2022, Tsvetkov et al. 2022, Wang et al. 2022). These 13 genes were FDX1, LIPT1, LIAS, DLD, DBT, GCSH, DLST, DLAT, PDHA1, PDHB, SLC31A1, ATP7A, and ATP7B. Based on the expression levels of the above 13 genes, we performed unsupervised clustering analysis in the training set to identify various clusters in LUAD using the package “ConsensusClusterPlus”. The number of clusters and patients in each subgroup was determined using the consensus cluster algorithm. A principal component analysis (PCA) was performed to test its stability.

### Gene set variation analysis (GSVA) and functional enrichment analysis

To further explore variations in biological processes between various clusters, we performed GSVA, which can detect slight differences in pathway activity over a sample population in an unsupervised manner using the “GSVA” package in R. We also conducted Gene Ontology annotation using the differentially expressed genes between the clusters using the “clusterProfiler” package in R. Pathways with *P* < 0.05 were considered to be significantly different between the clusters.

### Estimation of the immune cell infiltration profile in the tumor microenvironment (TME)

We conducted a single-sample gene set enrichment analysis (ssGSEA) algorithm to estimate the immune cell infiltration levels of 497 LUAD samples obtained from the TCGA dataset. The results were validated using CIBERSORT, which evaluates the relative proportion of 22 different immune cells in each sample based on their expression profiles using the leukocyte gene signature matrix LM22. Furthermore, the ESTIMATE algorithm was applied to calculate the estimated immune and stromal scores for various clusters. We employed the “ggpubr” package in R to present the differences between the clusters.

### Exploration of response to immune checkpoint inhibitors and drug sensitivity

To predict the effectiveness of immune checkpoint blockade (ICB) treatment, we applied the tumor immune dysfunction and exclusion (TIDE) algorithm (available at http://tide.dfci.harvard.edu) based on the expression profiles of patients with LUAD. The TIDE scores, as well as the infiltration levels of cancer-associated fibroblasts (CAFs), myeloid-derived suppressor cells (MDSCs), and M2 type tumor-associated macrophages, were calculated and compared between the clusters. The IMvigor210 cohort, an independent immunotherapeutic cohort, was used in this study. Expression data and the corresponding clinical information were obtained from http://research-pub.gene.com/imvigor210corebiologies. To investigate the chemotherapeutic response of the most commonly used drugs for Patients with LUAD, the “pRRophetic” package in R was used. The half-maximal inhibitory concentration (IC50) was compared based on the expression profile of Patients with LUAD and drug sensitivity data of LUAD cell lines, which were downloaded from the Genomics of Drug Sensitivity in Cancer (GDSC) database (www.cancerRxgene.org).

### Identification of differentially expressed genes (DEGs) between distinct subgroups

The “limma” package in R was applied to identify the DEGs between the subgroups under the criteria of adj. P value < 0.05 and the absolute value of fold change (|FC|) ≥ 2. Furthermore, univariate Cox regression analysis was conducted to determine the genes that were closely related to the overall survival time of patients with LUAD. Genes with P < 0.001 and a hazard ratio (HR) > 1 were selected as candidate genes for further analysis. The “survival” and “survminer” packages in R were used.

### Construction of the cuproptosis score

To assess cuproptosis level and predict the prognosis of each patient with LUAD, we developed a cuproptosis scoring scheme. Following univariate Cox regression analysis, genes with a significant prognosis were selected to conduct recursive feature elimination with random forest and tenfold cross-validation method using the “caret” package in R. As in previous studies, we defined the cuproptosis score (CPS) using the following formula:

$$CPS=\sum_{i=1}^{i=n}{C}_{i}\cdot {\nu }_{i}$$

where n denotes the number of mRNA in the model, c_i_ denotes the coefficient of the included mRNA, and v_i_ represents their expression level.

### Statistical analysis

All statistical analyses were conducted using the R software (version 4.0.1). The statistical significance of normally distributed variables was evaluated using the Student’s t test. The Kruskal–Wallis test was used to analyze the statistical significance of non-parametric comparisons of the three groups of variables. *P* < 0.05 was considered statistically significant, unless otherwise specified.

## Results

### Landscape of genetic variation of cuproptosis-related genes in LUAD

**Fig. 1:**
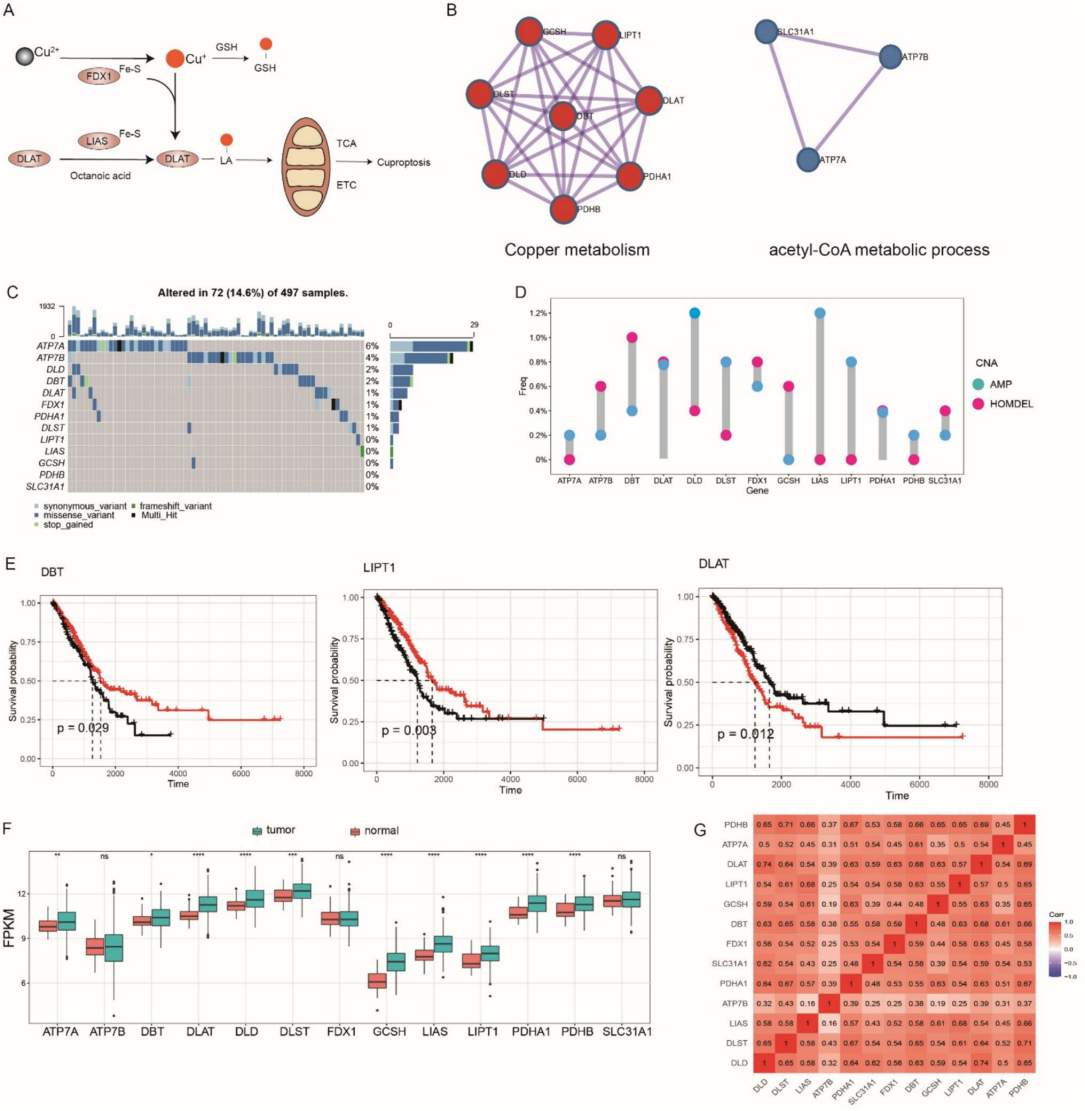
Multiple-omics landscape of cuproptosis-related genes in lung adenocarcinoma (LUAD). **A** Signaling and mechanism of cuproptosis. **B** Metascape enrichment analysis of the 13 cuproptosis-related genes. **C** Mutation frequency of 13 cuproptosis-related genes in 497 patients with LUAD from the TCGA-LUAD cohort. Each column represents an individual patient with LUAD. The upper bar plot represents TMB. The right-hand bar indicates the proportion of each variate type. **D** The CNV frequency of cuproptosis-related genes in the TCGA-LUAD cohort. The height of the column indicates the alteration frequency. Blue dots represent deletion frequency; red dots represent amplification frequency. **E** Kaplan–Meier curves of patients with LUAD from different risk groups divided by the expression profile of dihydrolipoamide S-acetyltransferase (DLAT), dihydrolipoamide branched chain transacylase E2 (DBT), and LIPT1. **F** Expression levels of 13 cuproptosis-related genes between LUAD tissues and normal tissues in the TCGA-LUAD cohort. Tumor, green; normal, red. (G) Correlation analysis of the 13 cuproptosis-related genes in LUAD

In this study, the roles of 13 cuproptosis-related genes (FDX1, LIPT1, LIAS, DLD, DBT, GCSH, DLST, DLAT, PDHA1, PDHB, SLC31A1, ATP7A, and ATP7B) were investigated in LUAD. Figure 1A shows a brief overview of the mechanism of cuproptosis, a newly proposed form of regulated cell death induced by intracellular copper (Cu). The 13 genes listed above were selected for Metascape enrichment analysis. As expected, the enrichment results mainly included “acetyl-CoA metabolic process’’ and “Copper metabolism”, which are both indispensable biological processes that mediate cuproptosis (Fig. 1B). We then investigated the frequency of copy number variations in these cuproptosis-related genes in LUAD. Genetic alterations were found to occur in 72 of the 497 LUAD samples, and missense mutations were the most common type of mutation (Fig. 1C). ATP7A showed the highest mutation frequency, followed by ATP7B, DLD, and DBT. We also evaluated CNV mutations in cuproptosis-related genes in Patients with LUAD (Fig. 1D, Supplementary Table 1). To explore whether these genetic alterations affect the expression of cuproptosis-related genes in patients with LUAD, we compared their mRNA expression levels between LUAD and normal samples in the TCGA-LUAD cohort and found that the majority of cuproptosis-related genes showed a significantly higher expression level in LUAD samples, except for ATP7B, FDX1, and SLC31A1 (Fig. 1F). Furthermore, the 497 patients with LUAD were divided into two groups based on the expression profiles of the 13 genes. We performed survival analysis between each group, and survival curves were compared using the log-rank test. Significant differences were found between groups stratified according to the expression profiles of DBT, DLAT, and LIPT1 (Fig. 1E). This suggests a close relationship between DBT, DLAT, and LIPT1 and the prognosis of patients with LUAD. To explore the relationship between cuproptosis-related genes in LUAD, we performed a co-expression analysis of these genes (Fig. 1G). It is worth mentioning that the DBT and DLAT levels were highly correlated.

**Fig. 2:**
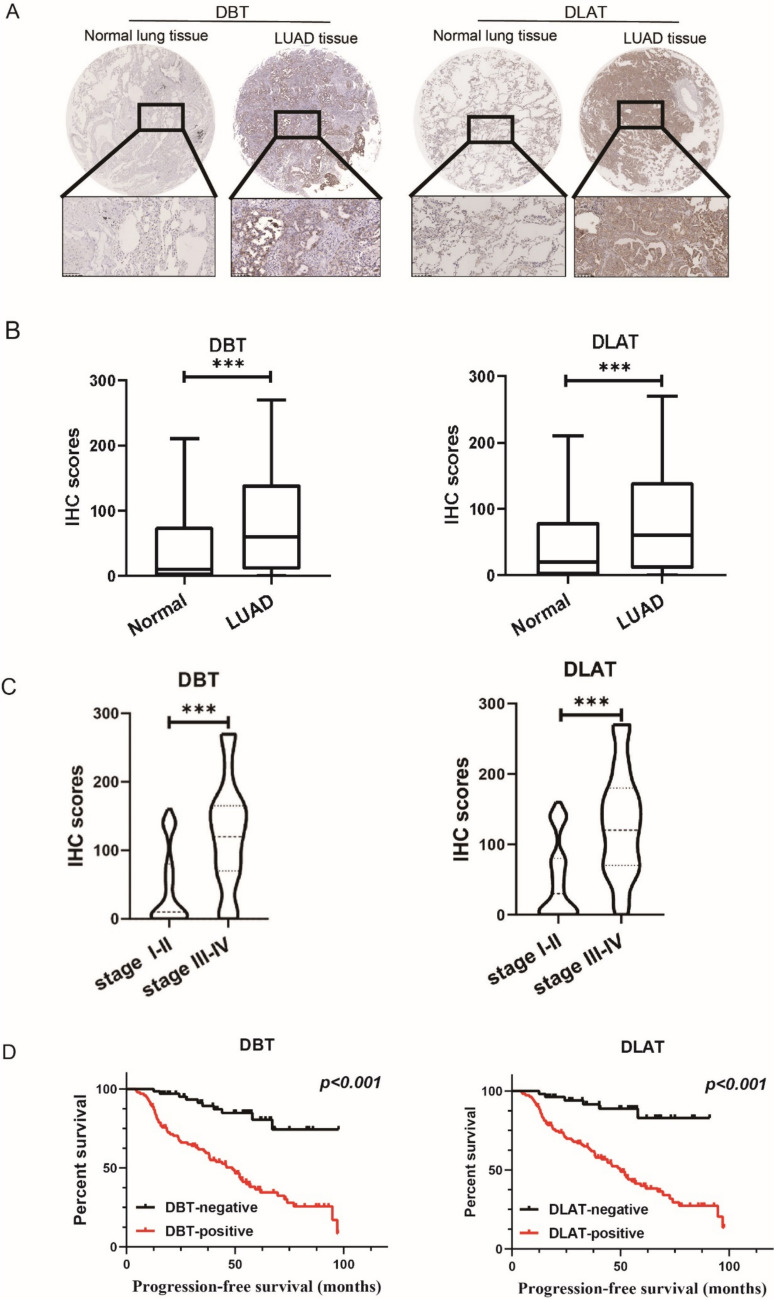
Immunohistochemistry (IHC) for DBT and DLAT was conducted on 165 normal lung and LUAD samples. **A** Representative IHC images showing the high expression levels of DBT and DLAT in LUAD and their low expression levels in normal lung tissues. **B** IHC scores of DBT and DLAT were significantly decreased in normal lung tissues compared to LUAD tissues. **C** IHC scores of DBT and DLAT were significantly decreased in LUAD tissues at early stages compared to LUAD tissues at advanced stages. **D** Kaplan–Meier survival analysis and log‐rank tests indicate that high levels of DLAT and DBT expression were associated with worse outcomes in LUAD (*P* < 0.001)

IHC staining analysis revealed that the expression levels of DLAT and DBT were elevated in LUAD tissues (n = 165) compared with those in their paired normal lung tissues (n = 165). Representative images of DLAT and DBT staining in normal lung and LUAD tissues are shown in Fig. 2A. Compared with that in normal lung tissue, the expression of DLAT and DBT was significantly upregulated in LUAD tissue samples (Fig. 2B). Moreover, the IHC scores of DLAT and DBT were significantly increased in patients with advanced TNM stages (Fig. 2C). Based on IHC scores, patients were divided into DBT-positive (n = 99) and DBT-negative (n = 66) groups. DBT‐positive patients exhibited a shorter progression-free survival (PFS) time than DLAT-negative patients (*p* < 0.001) (Fig. 2D). The patients were also divided into DLAT‐positive (n = 53) and DLAT‐negative (n = 112) groups. DLAT‐positive patients presented with a shorter progression-free survival time than DLAT‐negative patients (*p* < 0.001) (Fig. 2D). Univariate and multivariate analyses revealed that DLAT and DBT were independent factors for PFS in patients with LUAD. In contrast, the DBT‐positive (hazard ratio, 2.305; 95% confidence interval, 1.047–5.073; *p* = 0.038) and DLAT‐positive groups (hazard ratio, 2.832; 95% confidence interval, 1.080–7.425; *p* = 0.034) showed shorter PFS rates (Table 1). These data indicate that DLAT and DBT may represent new prognostic biomarkers for LUAD.

**Table 1:** Cox regression analysis of overall survival in LUAD patients

Variables	Univariate analysis			Multivariate analysis		
	HR	95% CI	P value	HR	95% CI	P value
Age (years)	0.972	0.947–0.999	0.040*	0.985	0.955–1.016	0.338
Gender (male vs female)	0.740	0.465–1.176	0.202	–	–	–
Smoking history	1.858	1.172–2.943	0.008*	1.863	1.124–3.088	0.016*
Differentiation	0.465	0.342–0.631	< 0.001*	0.586	0.409–0.839	0.003*
pT stage	1.496	1.181–1.896	0.001*	1.073	0.798–1.443	0.642
pN stage	2.394	1.498–3.827	< 0.001*	0.918	0.484–1.740	0.792
pM stage	1.416	0.760–2.637	0.273	–	–	–
pTNM stage	1.406	1.153–1.714	0.001*	1.119	0.826–1.516	0.468
DBT expression	4.985	2.556–9.722	< 0.001*	2.305	1.047–5.073	0.038*
DLAT expression	6.103	2.644–14.091	< 0.001*	2.832	1.080–7.425	0.034*

Factors that were significant are bolded and are defined as a P value < 0.05. Factors for which P < 0.05 in univariate analysis were subsequently used for multivariate analysis.

### Identification of two distinct LUAD patterns mediated by 13 cuproptosis-related genes

**Fig. 3:**
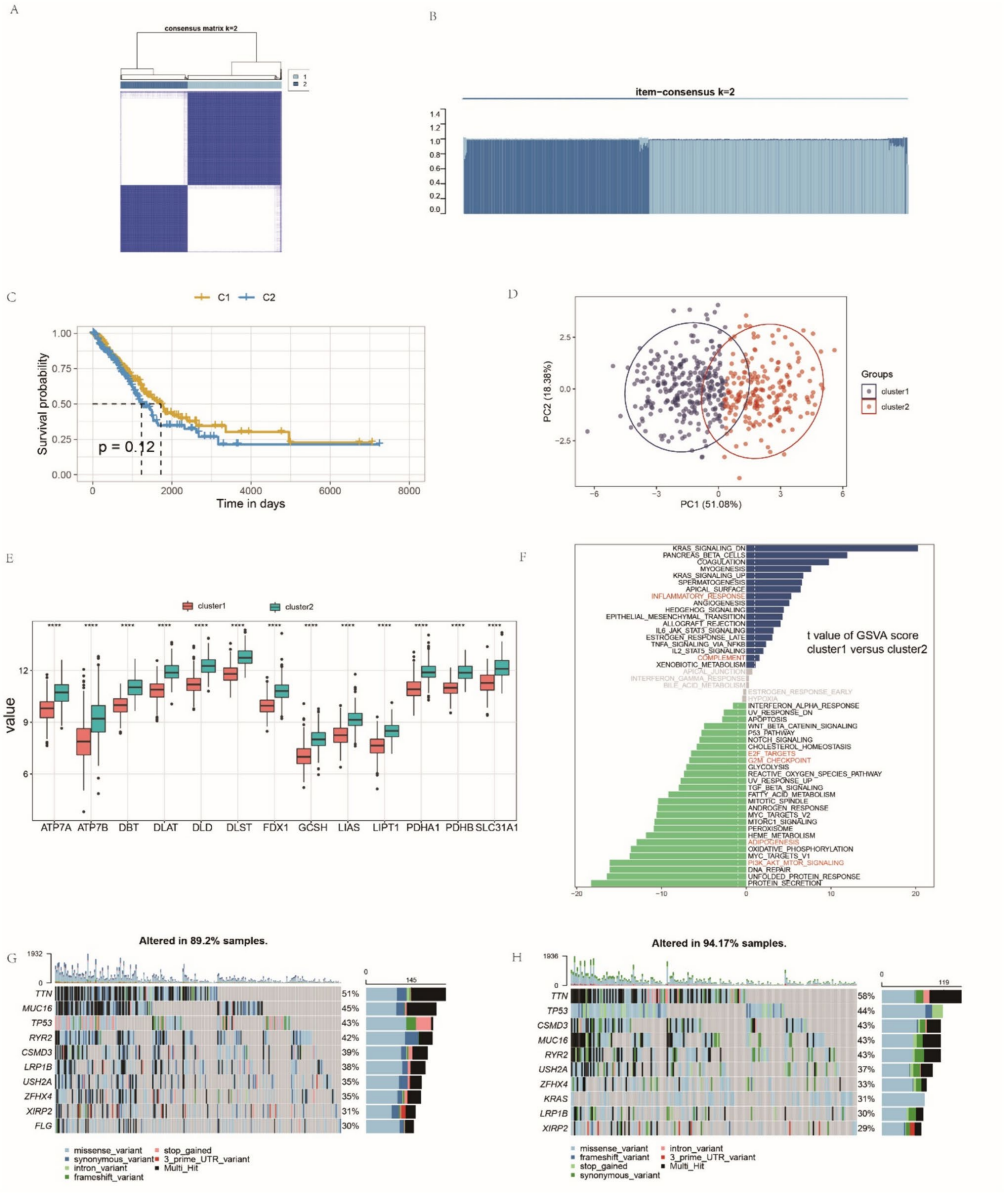
Identification of two distinct lung adenocarcinoma (LUAD) patterns mediated by 13 cuproptosis-related genes. **A**, **B** Similarity matrix of TCGA-Patients with LUAD derived from consensus clustering assays. **C** Comparison of the survival differences between the two identified cuproptosis-related subtypes using the log-rank test. **D** Principal component analysis of two distinct patterns in the TCGA-LUAD cohort. **E** Expression levels of 13 cuproptosis-related genes between cluster 1 and cluster 2. Cluster 1, orange; cluster 2, green. **F** The gene set variation analysis (GSVA) score of representative biological pathways in the two clusters are shown in the heatmap. (G-H) Mutation frequency of 13 cuproptosis-related genes in cluster 1 and cluster 2, respectively. Each column represents an individual patient with LUAD. The upper bar plot represents TMB. The right-hand bar indicates the proportion of each variate type

Regarding the importance of molecular subtypes in tumor treatment, especially immunotherapy, the “ConsensusClusterPlus” package in R, which is based on the algorithm of unsupervised consensus clustering, was applied to identify possible cuproptosis-related subtypes in LUAD based on the expression of 13 cuproptosis-related genes. Accordingly, after repeated subsampling and clustering, two distinct cuproptosis-related subtypes were identified: 290 samples in cluster 1 and 207 samples in cluster 2. Plots of the consensus matrix and item-consensus when k = 2 are shown (Fig. 3A, B). The cluster partition was “clean” and only a few cases had mixed cluster association, which indicates that the quantitative and visual stability were achieved. Next, survival analysis of the identified clusters was conducted; although not significant, cluster 1 exhibited a relatively better overall survival (Fig. 3C). The two clusters were also distinguished by applying PCA to 13 cuproptosis-related genes (Fig. 3D). A marked difference was observed in the expression of all 13 genes between different clusters; cluster 2 showed a higher expression level, which may indicate a more active cuproptosis level in the tumor microenvironment (Fig. 3E).

GSVA was used to evaluate discrepancies in pathway activity between the clusters (Fig. 3F) (Supplementary Table 2). As shown in the heatmap, cluster 1 was mainly enriched in pathways related to immune activation, such as the “INFLAMMATORY RESPONSE” pathway and the “COMPLEMENT” pathway. Compared with cluster 1, cluster 2 was particularly enriched in the “MITOTIC SPINDLE” pathway, as well as the “E2F TARGETS” pathway, which are closely related to the uncontrolled proliferation of cancer cells (Kent and Leone 2019; Sinha et al. 2019). It is also enriched in the “G2_M CHECKPOINT” pathway, which is an indispensable functional checkpoint for DNA repair, particularly in cancer cells (Matheson et al. 2016). Moreover, the “ADIPOGENESIS” pathway, which was correlated with unfavorable tumor microenvironment and worse survival, and the “P13/AKT/mTOR” pathway, which has been proved to have a strong relationship with proliferation, migration, and invasion, were also activated in cluster 2 (Gasparri et al. 2018; Oshi et al. 2021). The frequencies of copy number variations were also compared between the clusters (Fig. 3G, H). A large overlap in the top 10 high-frequency mutant genes of these two clusters was observed, which reflected the common mutation features of patients with LUAD.

### Characteristics of TME cell infiltration in different cuproptosis subtypes

**Fig. 4:**
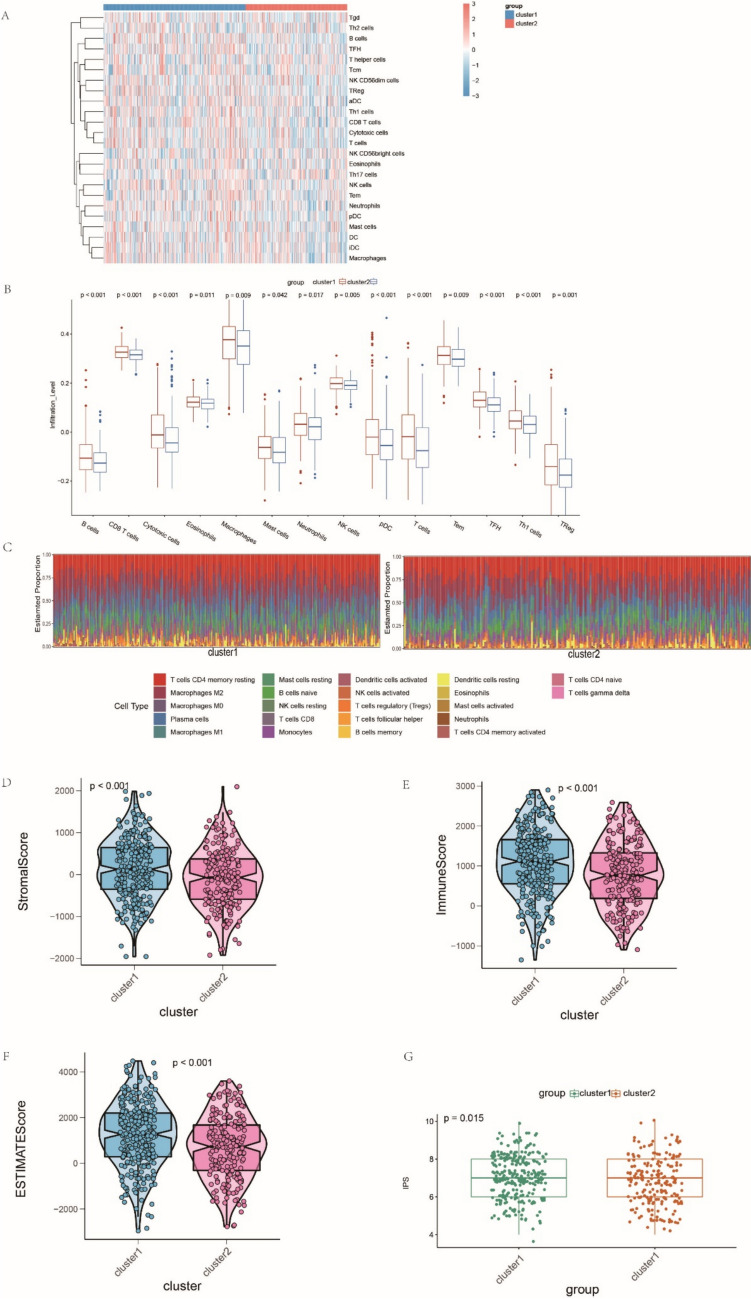
Characteristics of tumor microenvironment (TME) cell infiltration in different cuproptosis-related subtypes. **A** The heatmap shows the immune infiltration level of 24 cell types evaluated by single-sample gene set enrichment analysis (ssGSEA) analysis. **B** Comparison of the infiltrated proportion of representative immune cells between the subtypes. **C** Histograms display the immune landscape in different clusters estimated by CIBERSORT. **D**–**F** The relative distribution of the stromal score, immune score, and ESTIMATE score between the clusters. **G** The relative distribution of immunophenoscore (IPS) in the two subtypes

To evaluate the difference in TME infiltration between the subtypes, we first employed ssGSEA, a commonly used algorithm based on integrated immune gene sets from published studies, to determine their immune infiltration levels. Compared with cluster 2, we noticed a relatively higher overall immune level in the TME of cluster 1 from the heatmap (Fig. 4A). Figure 4B shows the detailed discrepancy in abundance levels and the proportion of 14 types of immune cells; cluster 1 exhibited a significantly higher abundance of various immune cell types, including NK cells, mast cells, neutrophils and eosinophils, pDCs, Tem cells, TFH cells, Tregs, and B cells. The results of the CIBERSORT analysis, a deconvolution algorithm that could quantify the immune composition of tumor samples, also confirmed that clusters 1 and 2 had distinct immune profiles (Fig. 4C). Based on the above findings and previous studies, we found that these two cuproptosis subtypes in LUAD are consistent with the previously presented immune-based classification of tumors. Cluster 1 can be classified as the immune-hot phenotype, which is characterized by tumor-infiltrating lymphocyte (TILs) infiltration and antitumor response, whereas cluster 2, which has a relatively higher level of cuproptosis, can be classified as the immune-cold phenotype, characterized by immunological ignorance. The results of the ESTIMATE analysis further confirm our assumption. As expected, the immune-hot phenotype had a significantly higher immune score, which represents the infiltration of immune cells, than the immune-cold phenotype did. Significant differences were also found in the stromal and estimated scores, with a higher stromal score indicating a greater abundance of stroma in the immune-hot phenotype. The estimated score, which indicates the abundance of tumor cells in the TME, also exhibited a higher level of the immune-hot phenotype (Fig. 4D–F).

**Fig. 5:**
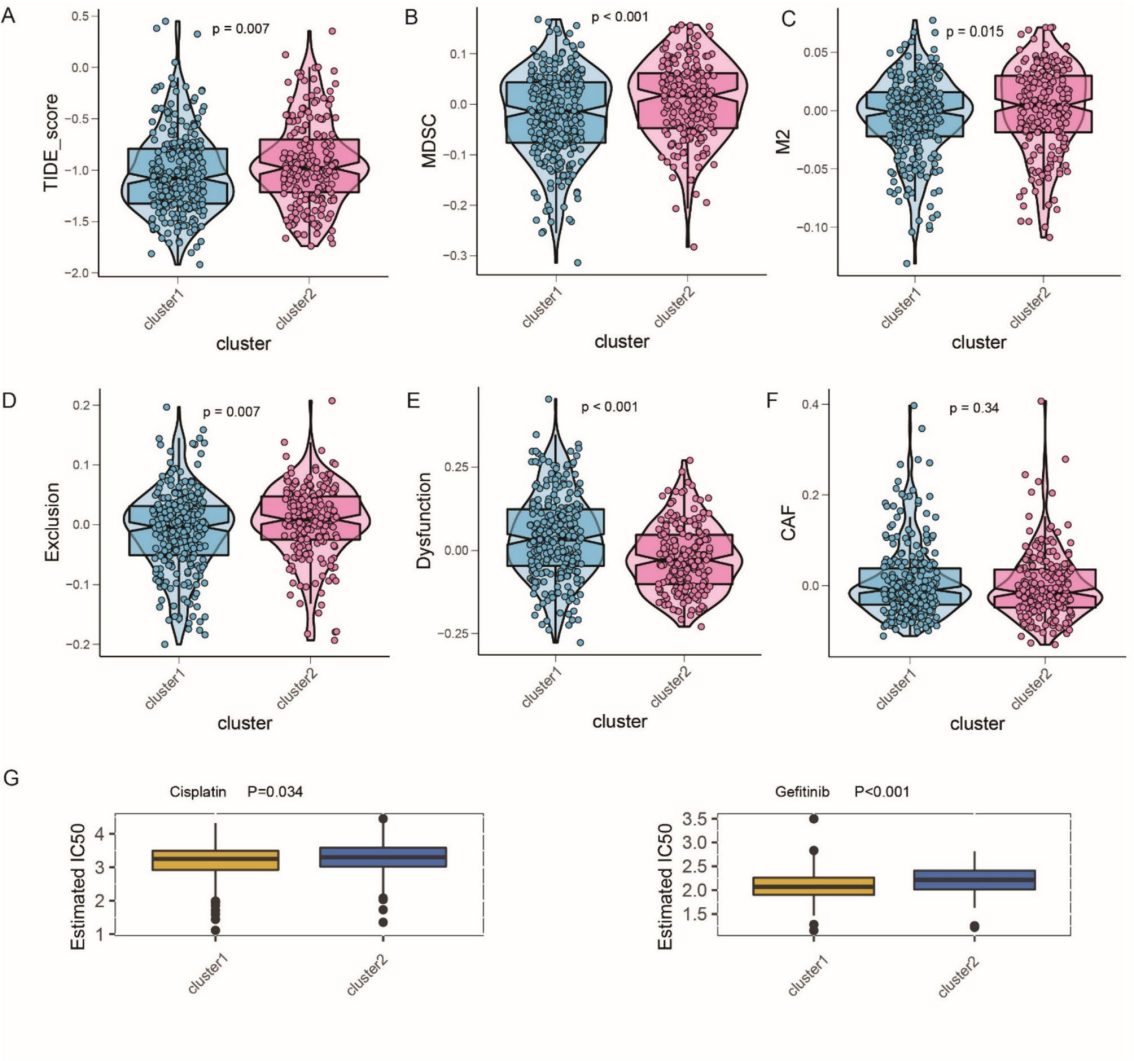
Differences in immunotherapeutic benefits and drug sensitivity between the two cuproptosis-related subtypes. **A** The relative distribution of the tumor immune dysfunction and exclusion (TIDE) score between the subtypes. **B** The relative distribution of the proportion of myeloid-derived suppressor cells (MDSCs) in TME between the subtypes. **C** The relative distribution of the proportion of M2-macrophage in TME between the subtypes. **D** The relative distribution of the proportion of the exclusion score calculated by TIDE between the subtypes. **E** The relative distribution of the dysfunction score calculated by TIDE between the subtypes. **F** The relative distribution of the proportion of cancer-associated fibroblasts (CAFs) in TME between the subtypes. **G** The differences in drug sensitivity of the commonly used drugs between distinct lung adenocarcinoma (LUAD) patterns

To better understand the discrepancy in cuproptosis subtypes, we investigated their response to immunotherapy and their diversity in drug resistance. To this end, we first compared their immunophenoscore (IPS), which was calculated using a machine learning algorithm based on the gene expression profile. A statistically higher IPS in cluster 1 indicated a better response to immunotherapy (Fig. 4G). TIDE was also conducted, which is a reliable computational framework for predicting ICB response and uncovering the mechanism of immune escape. The TIDE scores of patients with LUAD in TCGA cohort were calculated, and the discrepancies between the two clusters were compared. Consistent with the above results, a higher TIDE score was observed in cluster 2, indicating a lower response rate to ICB treatment (Fig. 5A). Cluster 2, which we termed the immune-cold phenotype, also exhibited significantly higher levels of cell types that have been reported to restrict T-cell infiltration in the TME, including MDSCs and M2 subtype tumor-associated macrophages (Fig. 5B, C). However, the CAFs did not show a significant difference between the clusters (Fig. 5F). Significantly higher TIDE exclusion scores in cluster 2, which had a strong negative correlation with the infiltration level of cytotoxic T lymphocytes, also showed lower ICB effectiveness in cluster 2 (Fig. 5D). A relatively higher T cell dysfunction score was also observed in cluster 1 (Fig. 5E). Furthermore, based on the GDSC database, we explored the sensitivity of the most frequently used anti-LUAD drugs and estimated the half-maximal inhibitory concentration (IC50) for each patient with LUAD in the training cohort. As expected, significant differences were observed between the two clusters. Cisplatin and gefitinib exhibited relatively better responses in cluster 1 (Fig. 5G).

### Construction of CPS and evaluation of its role in prognosis, clinical relevance, and anti-PD-L1 immunotherapy

**Fig. 6:**
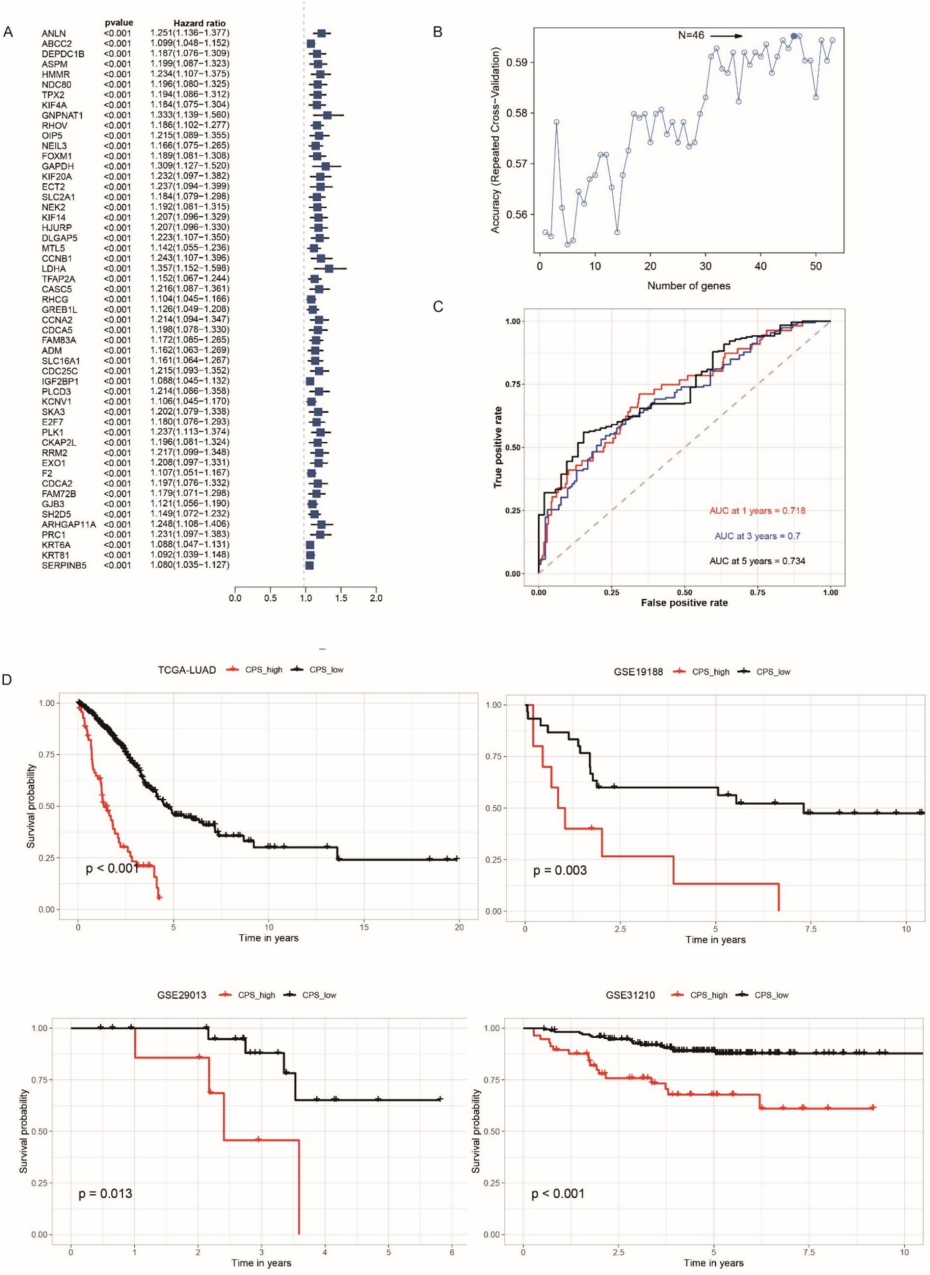
Construction and validation of the prognosis predicting function of cuproptosis score (CPS). **A** The P value and hazard ratio of the genes that had a significant relationship with the overall survival of patients with lung adenocarcinoma (LUAD). **B** Accuracy of repeated cross-validation. **C** Area under the curve (AUC) of the CPS in predicting the 1-, 3-, and 5-year survival of patients with LUAD. **D** Survival analysis based on the established CPS was performed using the log-rank test

To better understand the characteristics of cuproptosis in patients with LUAD, 1842 DEGs between the two cuproptosis subtypes identified above were obtained under the criteria of adj. P-value < 0.05, and |FC|≥ 2, and further subjected to univariate Cox regression analysis. A total of 53 genes were screened out with the criteria of *P* < 0.05 and hazard ratio (HR) > 1 (Fig. 6A, Supplementary Table 3). After confirming the strong relationship between cuproptosis and immune infiltration, we constructed a scoring scheme called CPS to predict prognosis and evaluate the level of cuproptosis in each patient with LUAD. Genes proven to have a significant relationship with prognosis by univariate regression analysis were subjected to a recursive feature elimination algorithm and repeated cross-validation. Finally, 46 genes were selected and a scoring scheme was constructed (Fig. 6B). The CPS of patients with LUAD in the training set was calculated by combining their expression levels in each individual patient with LUAD and the coefficients of the 46 genes (Supplementary Table 4). Receiver operating characteristic curve analysis was conducted to evaluate the effectiveness of the cuproptosis signature. The signature was proven to have a good prognosis predicting performance, with 1-, 3-, and 5-year areas under the curve (AUC) of 0.718, 0.700, and 0.734, respectively (Fig. 6C). Based on the optimal cut-off point, 497 patients with LUAD were divided into high- and low-CPS groups. As shown in the Kaplan–Meier survival curve, significantly better OS was observed in the low-risk group. In addition to the 497 patients with LUAD in our training set, we included 296 patients with LUAD from three independent GSE LUAD cohorts to test the robustness of this CPS. The high-risk and low-risk groups, which were divided using the same formula, exhibited remarkably different mortality rates (Fig. 6D). These results further verified the stability and accuracy of the CPS in evaluating the prognosis of patients with LUAD.

**Fig. 7:**
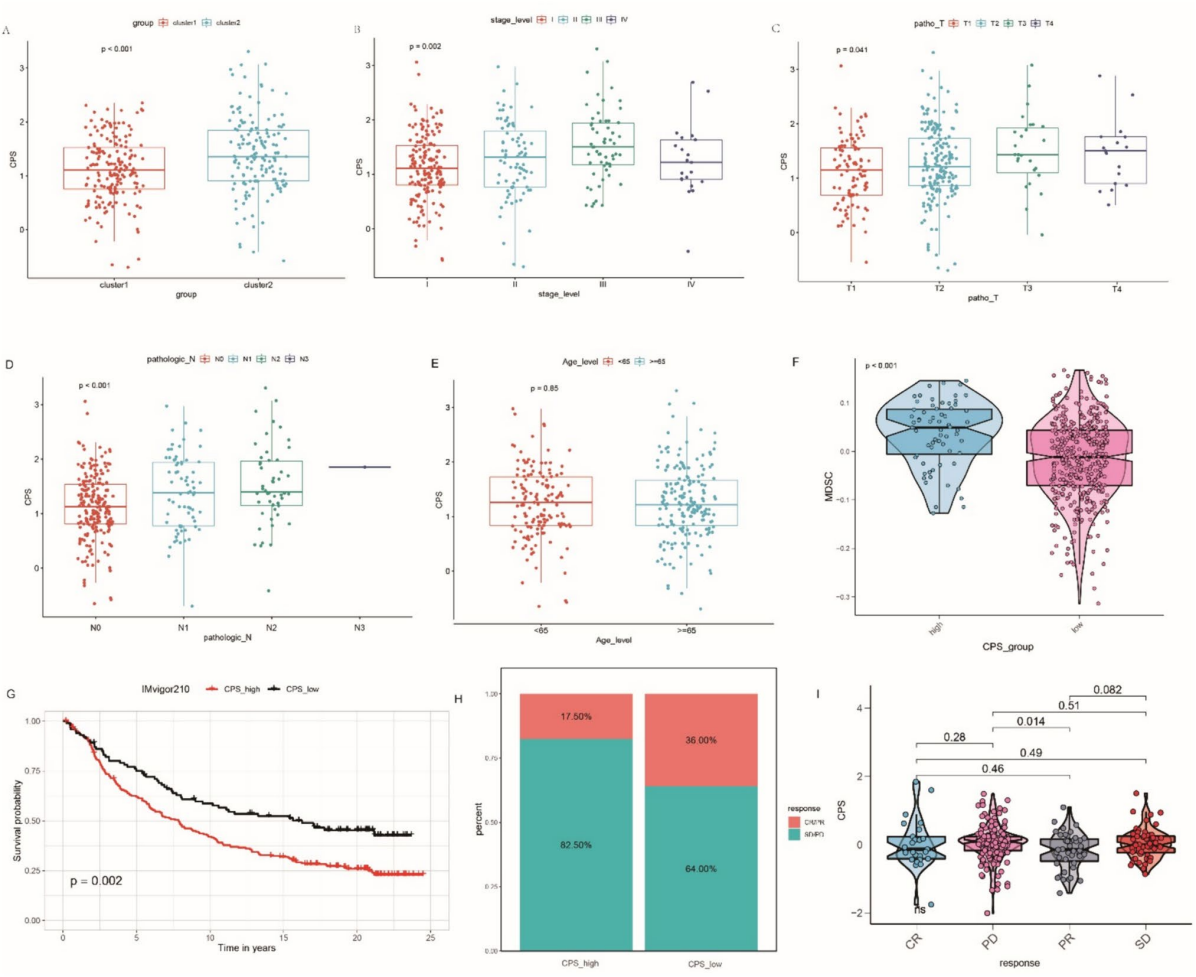
Exploration of the clinical relevance of cuproptosis score (CPS). **A** Relative distribution of the CPS between cluster 1 and cluster 2. **B** Relative distribution of the CPS among patients with LUAD of different stages. **C** Relative distribution of the CPS among the patients with LUAD of tumor size. **D** Relative distribution of the CPS among the patients with LUAD of different extents of lymph node metastases. **E** Relative distribution of CPS between patients with LUAD less than and older than 65 years. **F** Relative distribution of myeloid-derived suppressor cells (MDSCs) between the low- and high-CPS groups. **G** Comparison of the survival differences between the low- and high-CPS groups of the anti-PD-L1 immunotherapy cohort using the log-rank test. **H** The proportion of patients’ response rate to anti-PD-L1 immunotherapy in the low- and high-CPS groups. *CR* complete response, *PR* partial response, *SD* stable disease, *PD* progressive disease. **I** Distribution of CPSs in different anti-PD-L1 response groups

We also explored the relationship between CPS and the two cuproptosis subtypes. Cluster 1, which had a better prognosis and higher immune infiltration, tended to have a lower CPS than cluster 2 (Fig. 7A). This suggests that CPS is closely associated with the cuproptosis subtype and survival outcomes of patients with LUAD. Additionally, we investigated the clinical relevance of the CPS in patients with LUAD. Compared with that in early stage LUAD, a pronounced elevation in the CPS was observed in advanced-stage LUAD. However, the tendency of stage IV was not obvious which may be affected by the limited number of patients in this group (Fig. 7B). Similarly, the larger the tumor size and the more extensive the extent of lymph node metastases, the higher was the CPS received (Fig. 7C, D). No significant relationship was observed between the age of the patients with LUAD and their CPS (Fig. 7E). MDSCs, which are recognized as immunosuppressive components in tumors, were also abundantly infiltrated in the CPS-low group (Fig. 7F).

We further used the anti-PD-L1 cohort (IMvigor210) to explore the role of CPS in immunotherapy. Consistent with the findings above, significantly prolonged overall survival was observed in the group with a lower CPS (Fig. 7G). In addition, the proportion of patients in the lower CPS group who had a complete or partial response to anti-PD-L1 immunotherapy (36%) was considerably higher than that of the higher CPS group (17.5%) (Fig. 7H). Among the different anti-PD-L1 clinical response groups, the complete response group had the lowest CPS, whereas the progressive disease group had the highest score (Fig. 7I). These results confirm that patients with a lower CPS may have a better prognosis and response to immunotherapy. The cuproptosis scoring scheme established in this study may contribute to predicting prognosis and response to anti-PD-L1 immunotherapy.

## Discussion

In this study, DLAT and DBT significantly correlated with LUAD occurrence and prognosis. Two distinct cuproptosis-related patterns were observed in LUAD. We further developed a scoring scheme called the CPS to quantify the cuproptosis-related pattern of each individual patient with LUAD. Patients with a lower CPS were shown to have a relatively better prognosis, more abundant immune infiltration in the TME, and an enhanced response to immune checkpoint inhibitors.

First, we explored the biological functions of the cuproptosis-related genes identified in LUAD. The results of survival analysis revealed a significant close relationship between the prognosis of patients with LUAD and the expression profiles of DBT, DLAT and LIPT1 in LUAD. And co-expression analysis of the above three genes indicated that the correlation coefficient of DLAT and DBT was relatively higher than that of LIPT1 and DBT, LIPT1 and DLAT. DBT and DLAT, which are essential components of the PDH complex, are two essential enzymes in protein lipoylation that play an indispensable role in the process of cuproptosis. The relationships between DLAT and various types of tumors have been extensively studied. It has been found that mRNA levels of DLAT are significantly higher in tumor tissues of liver hepatocellular carcinoma and stomach adenocarcinoma compared to normal tissues, whereas they are lower in head and neck squamous cell carcinoma and kidney renal clear cell carcinoma (Yang et al. 2023). DLAT expression has also been identified as a prognostic factor in several cancers, where higher levels are associated with poorer survival outcomes (Xu et al. 2023). Furthermore, the expression levels of DLAT correlate with responses to chemotherapeutic agents like gemcitabine and oxaliplatin, suggesting its potential as a biomarker for chemoresistance in pancreatic cancer (Fang et al. 2023). DLAT imbalance influences cell proliferation and migration, and silencing DLAT has been shown to increase pyruvate levels and reduce cancer growth in gastric cancer (Goh et al. 2015). However, the relationship between DBT and tumors is yet to be extensively studied. DBT has been characterized as a tumor suppressor that inhibits tumor progression and corrects lipid metabolism disorders through the DBT/ANXA2/YAP axis-regulated Hippo signaling pathway in clear cell renal cell carcinoma (Miao et al. 2023). DBT expression has also been recognized as an independent prognostic factor in kidney renal clear cell carcinoma, highly correlated with immunological checkpoints and targeted medications (Zhang et al. 2023). In this study, a strong positive correlation was observed between DLAT and DBT expression levels in patients with LUAD. Compared with normal lung tissues, significant upregulation of DLAT and DBT was observed in LUAD tissues. Moreover, significant correlations were observed between the expression profiles and survival time of patients with LUAD. Consistent with these results, high DLAT expression was previously found to enhance the progression of non-small cell lung cancer via DLAT-mediated glycolysis (Chen et al. 2022). DLAT also functions as an acetyltransferase necessary for 6PGD lysine acetylation, which is of vital importance for tumor growth (Shan et al. 2014). Interestingly, in our study, correlation analysis revealed a negative correlation between immune cell infiltration and the expression levels of DLAT and DBT, suggesting that cuproptosis-related genes such as DLAT and DBT may promote the invasion of cancer cells through immunosuppression. Furthermore, as previously mentioned, both enzymes are essential components of the PDH complex, which plays a critical role in converting pyruvate to acetyl-CoA, thus bridging glycolysis and the citric acid cycle. This pathway is commonly exploited by cancer cells to satisfy their heightened metabolic needs. Therefore, the involvement of these enzymes in LUAD may stem from their fundamental roles in cellular metabolism, which is frequently reprogrammed in LUAD cells. While direct studies connecting DBT and DLAT mutations to cancer are scarce, the significant role these enzymes play in metabolic pathways suggests they could impact LUAD development and progression by causing metabolic dysregulation. Further experimental validation is required to determine specific mechanisms.

Thus, in this study, we first discovered two distinct subtypes of LUAD based on 13 cuproptosis-related genes summarized in the literature. We found substantial differences in the TME characteristics and immune landscapes between the two subtypes of LUAD. ssGSEA and CIBERSORT were used to evaluate TME cell infiltration. Compared with cluster 2, a relatively higher immune score, which represented a higher immune infiltration level in the TME, was found in cluster 1. Cell types, such as NK cells, Tregs, and B cells, which mainly infiltrated cluster 1, have the potential to promote anti-tumor immunity and be targeted for cell-based therapies (Whiteside 2018; Terren et al. 2019; Rubio et al. 2020). Additionally, mast cells, neutrophils, eosinophils, pDCs, Tem cells, and TFH cells all showed significantly higher levels of infiltration in cluster 1. Tumor-associated mast cells have been associated with improved overall survival (OS) in NSCLC, regardless of stage(Shikotra et al. 2016). Human neutrophils have been identified as releasing ELANE, which can selectively target and destroy a wide range of cancer cells (Cui et al. 2021). Plasmacytoid dendritic cells (pDCs), recognized as major producers of type I interferons, exhibit anti-tumor effects(Reizis 2019). Furthermore, effector memory T cells (Tem) provide rapid responses to antigens they have previously encountered, potentially enhancing tumor surveillance. The heterogeneity in immune cell infiltration could be indicative of differing immune evasion strategies employed by the tumor cells within these LUAD samples. A robust immune cell presence in cluster 1 indeed correlates with better prognosis and response to immunotherapies, like checkpoint inhibitors that aim to reinvigorate the immune system against the tumor. Nevertheless, cluster 2 was abundantly infiltrated with myeloid-derived suppressor cells (MDSCs) and M2 subtype tumor-associated macrophages, which are recognized as immunosuppressive components in tumors and have a close connection with tumor progression (Mantovani et al. 2002; Kumar et al. 2016). Accordingly, cluster 1 also had a relatively better prognosis. After comparing their TME characteristics and referring to previous studies, we termed the two clusters as “immune-hot phenotype” and “immune-cold phenotype” respectively (Duan et al. 2020; Zhang et al. 2021a, b). Cluster 1, the immune-hot phenotype in LUAD, is characterized by TILs infiltration and anti-tumor response. On the other hand, cluster 2, the immune-cold phenotype in LUAD, was characterized by immunological ignorance. We further examined the discrepancy in the response rates to different types of immunotherapy and drug resistance using the TIDE and GDSC databases. Similarly, the immune hot phenotype of LUAD shows a higher response rate. Regarding drug sensitivity characteristics, significant differences were observed between the two subtypes. The immune hot phenotype was more sensitive to cisplatin and gefitinib. In summary, the two subtypes of LUAD, which were identified using cuproptosis-related genes, varied greatly in the immune landscape, drug resistance and sensitivity, and prognosis. We assert that cuproptosis contributes to the heterogeneity of LUAD and has the potential to guide individualized therapies for patients with LUAD.

We further constructed a scoring scheme called the CPS to quantify the cuproptosis level of each patient with LUAD more precisely and to explore its clinical relevance in LUAD. The immune-hot phenotype exhibited a lower CPS, whereas the immune-cold phenotype exhibited a higher CPS. Further analyses revealed that CPS was strongly associated with the clinical features of LUAD; additionally, it performed better in predicting prognosis using AUC as the reference index. CPS is positively correlated with the tumor stage of LUAD, which usually includes tumor size and the extent of lymph node metastases (Pineros et al. 2019). This indicated that the higher the CPS a patient with LUAD obtained, the worse the prognosis they obtained, which was also proven by survival analysis.

Due to the efficacious and durable response observed in clinical trials and patients with tumors, immunotherapy remains an optimistic therapy for lung cancer (Steven et al. 2016). Predicting their response to immunotherapy could maximize the clinical and economic benefits for patients with LUAD. In this study, by applying the same formula, patients with tumors in an anti-PD-L1 cohort (IMvigor210) were divided into high- and low-CPS groups. A significantly prolonged survival was observed in the low CPS group. More importantly, the response rate to anti-PD-L1 immunotherapy was markedly elevated in the low-CPS group. These findings further verified our hypothesis that CPS, which is closely related to anti-tumor immunity, is not only a prognostic biomarker in LUAD, but also a response predictor for immunotherapies. These results preliminarily revealed the network among cuproptosis, antitumor immunity, and LUAD.

This study had some limitations. Although the study included the retrospective data of 793 Patients with LUAD from the TCGA-LUAD database and several GSE datasets, more data are needed to verify the robustness of the CPS. In addition, the predictive ability of CPS for immunotherapy in LUAD has been indirectly verified. Therefore, an independent immunotherapy cohort for LUAD is required. The mechanism underlying the close connection between cuproptosis and LUAD requires further experimental verification.

## Conclusions

Cuproptosis has been found to influence the TME characteristics and heterogeneity in LUAD. Patients with a lower CPS had a relatively better prognosis, more abundant immune infiltration in the TME, and an enhanced response to immune checkpoint inhibitors.

### Supplementary Information

Below is the link to the electronic supplementary material.

Supplementary file1 (CSV 1 KB)

Supplementary file2 (CSV 5 KB)

Supplementary file3 (CSV 3 KB)

Supplementary file4 (CSV 1 KB)

Supplementary file5 (XLSX 39 KB)

## Data Availability

The datasets used and/or analysed during the current study are available from the corresponding author on reasonable request.
